# Resolutions Complimentary to Corydon La Ford, M. D., Demonstrator of Anatomy in the Medical Department of the University of Buffalo

**Published:** 1847-06

**Authors:** J. M. Hardy, Sidney A. Foss

**Affiliations:** President; Secretary


*Resolutions complimentary to Corydon La Ford, M. D., Demonstrator of Anatomy in the Medical Department of the University of Buffalo.*

We take pleasure in complying with the request to insert the following resolutions of the class now in attendance at the Medical College in this city.

At a meeting of the class, Med. Dep. University of Buffalo, held April 21, 1847, John M. Hardy was called to the chair, and S. A. Foss elected Secretary.

On motion, Messrs. Cary, Ely, White, Foss, and Hardy, were appointed a Committee to draft Resolutions expressing the sentiments of the Class, in regard to the *Anatomical Demonstrations*, and *Recapitulatory Lectures* just completed by CORYDON LA FORD, M. D.

The Committee reported the following Resolutions, which were unanimously adopted.

*Resolved*, That the able, interesting, and highly instructive course of Anatomical Lectures and Demonstrations, recently concluded by Dr. Corydon La Ford, meets the entire approbation of the Class; and, that the signal ability with which he imparted to us his scientific, and minute researches, excite our highest admiration.

*Resolved*, That as a *Demonstrator of Anatomy*, Dr. La Ford, is, in our opinion, *unsurpassed* by any of his age and experience; and in wishing him a more extended field of usefulness and influence we *could not ask any which he does not merit.*

*Resolved*, That the members of the Class entertain for Dr. La Ford the most profound esteem, for his scientific attainments; that they not only admire his zeal and industry in the prosecution of his favorite science, but, also, for those *moral qualities of the heart*, which shine forth so *conspicuously* in *his character.*

*Resolved*, That Dr. La Ford for his courteous and gentlemanly deportment, to us individually and collectively, merits our warmest thanks, and best wishes; and that he shall ever receive from us the tribute due from *grateful hearts.*

On motion of Mr. Blake—*Resolved*, That a copy of the proceedings of this meeting be forwarded to Dr. La Ford, and that they be published in "The Buffalo Medical Journal and Monthly Review of Medical and Surgical Science," and in "The New-York Journal of Medicine and Collateral Sciences.

J. M. HARDY, *President.*

SIDNEY A. FOSS, *Secretary.*

